# Autophagy and ubiquitin-dependent proteolysis processes in left ventricular mass loss in pulmonary arterial hypertension

**DOI:** 10.1038/s41598-024-64950-4

**Published:** 2024-07-02

**Authors:** Mateusz K. Hołda, Urszula Raźny, Maria Sordyl, Joanna Góralska, Maria Kapusta, Krystyna Słowińska-Solnica, Dorota Wojtysiak, Grzegorz Lis, Bogdan Solnica, Grzegorz Kopeć, Jakub Hołda

**Affiliations:** 1 HEART - Heart Embryology and Anatomy Research Team, Department of Anatomy, Jagiellonian University Medical College, Kopernika 12, 31-034 Kraków, Poland; 2 Division of Cardiovascular Sciences, The University of Manchester, Manchester, UK; 3 Department of Clinical Biochemistry, Jagiellonian University Medical College, Kraków, Poland; 4 Department of Animal Genetics, Breeding and Ethology, University of Agriculture in Cracow, Kraków, Poland; 5 Department of Histology, Jagiellonian University Medical College, Kraków, Poland; 6 Department of Cardiac and Vascular Diseases, Jagiellonian University Medical College, Kraków, Poland

**Keywords:** Pulmonary hypertension, Monocrotaline-induced PAH, Cardiac remodeling, Left ventricle atrophy, Cardiac autophagy, Ubiquitin–proteasome system, Cardiovascular biology, Anatomy

## Abstract

The goal of this study was to evaluate the intensity of autophagy and ubiquitin-dependent proteolysis processes occurring in myocardium of left ventricle (LV) in subsequent stages of pulmonary arterial hypertension (PAH) to determine mechanisms responsible for LV mass loss in a monocrotaline-induced PAH rat model. LV myocardium samples collected from 32 Wistar rats were analyzed in an early PAH group (n = 8), controls time-paired (n = 8), an end-stage PAH group (n = 8), and their controls (n = 8). Samples were subjected to histological analyses with immunofluorescence staining, autophagy assessment by western blotting, and evaluation of ubiquitin-dependent proteolysis in the LV by immunoprecipitation of ubiquitinated proteins. Echocardiographic, hemodynamic, and heart morphometric parameters were assessed regularly throughout the experiment. Considerable morphological and hemodynamic remodeling of the LV was observed over the course of PAH. The end-stage PAH was associated with significantly impaired LV systolic function and a decrease in LV mass. The LC3B-II expression in the LV was significantly higher in the end-stage PAH group compared to the early PAH group (*p* = 0.040). The measured LC3B-II/LC3B-I ratios in the end-stage PAH group were significantly elevated compared to the controls (*p* = 0.039). Immunofluorescence staining showed a significant increase in the abundance of LC3 puncta in the end-stage PAH group compared to the matched controls. There were no statistically significant differences in the levels of expression of all ubiquitinated proteins when comparing both PAH groups and matched controls. Autophagy may be considered as the mechanism behind the LV mass loss at the end stage of PAH.

## Introduction

Pulmonary arterial hypertension (PAH) is a rare, chronic, and life-threatening condition characterized by a progressive increase of pulmonary vascular resistance that leads to heart failure^1^. The annual incidence of PAH is approximately 2–7 new cases per million adults^2–4^. The signs and symptoms of PAH develop slowly and are non-characteristic^5^. Although challenging, early diagnosis and prompt treatment are important in managing PAH and improving patients’ quality of life^6^.

Development of right ventricular and later left ventricular heart failure secondary to PAH is a strong negative predictive factor. Several morphological changes of the heart secondary to chronically elevated blood pressure in the pulmonary vasculature are observed^7^. Knowledge on the sequence of cardiac morphological changes during the development of PAH may allow us to determine high-risk PAH patients and facilitate decisions about PAH targeted therapies.

Right ventricle hypertrophy occurs as an adaptive response to an increased right-ventricular afterload^8,9^. As PAH progresses, the compensatory factors collapse, followed by failure of the right ventricle induced by pressure overload^7,8^. The right ventricle’s functional and morphological failure develops progressively from the primary stage of PAH, remodeling of the left ventricle develops at the final stages of PAH^7,10,11^. Decreases in left ventricle mass, wall thickness, and stricture of its cavity occur at the end stages of the disease^7,11^. Furthermore, a gradual decrease of the left ventricle systolic pressure occurs^7,12^.

In recent years, there has been significant progress in the understanding of the underlying pathomechanisms of PAH, including molecular processes underlying heart remodeling over its course^7,13,14^. Although the mechanism of right ventricular remodeling and failure are attracting much attention among researchers, there is still incomplete familiarity with the basis of left ventricular mass loss and dysfunction^15,16^. Our recent shotgun proteomic study identified several significant alterations in the left ventricle that may be linked to PAH-induced left ventricular failure. For example, an increase in the apoptotic pathway activity was discovered^17^.

Autophagy is a process that plays a significant role in degradation of cardiomyocytes in response to hemodynamic stress^18^. Thus, participation of autophagy in the processes of the myocardial response to conditions of hemodynamic load may also occur in the case of PAH. Crosstalk occurs between the two synergistic degradation systems of autophagy and ubiquitin-dependent proteolysis, so the two mechanisms may synergistically complement each other to maintain cardiac cellular homeostasis^19^. Therefore, the goal of this study was to evaluate the intensity of autophagy and ubiquitin-dependent proteolysis processes occurring in the myocardium of the left ventricle at subsequent stages of PAH to determine the mechanisms responsible for left ventricular mass loss.

## Material and methods

### Animal model and experiment structure

This study is a part of the “Autophagy and ubiquitin–proteasome system as potential mechanisms responsible for left ventricle mass loss in a monocrotaline pulmonary hypertension rat model” project founded by the National Science Centre, Poland (2016/23/N/NZ5/00597). The animal model developed in this study was used also for other analyses^7,17^. The study was performed in accordance with the guidelines of Directive 2010/63/EU of the European Parliament on the protection of animals used for scientific purposes and was approved by the 2nd Local Ethical Committee in Cracow, Poland (No 60/2016). The study is reported in accordance with ARRIVE guidelines. A total of 66 Wistar male rats (8 weeks old; provided by Experimental Medicine Center of the Medical University of Bialystok, Poland) were randomly assigned to two groups. In the study group, 48 rats were injected intraperitoneally with a single dose of 60 mg/kg body weight of monocrotaline in Dulbecco’s phosphate-buffered saline (PBS) medium (3 mL/kg of body weight, Sigma-Aldrich, Germany) to induce PAH^20^. In the control group, animals (n = 18) were injected with the medium Dulbecco’s phosphate-buffered saline (PBS) medium (3 mL/kg of body weight, Sigma-Aldrich, Germany) without monocrotaline.

The rats were maintained under standard conditions and were fed a normal rat diet. They were subjected to regular transthoracic echocardiogram (TTE) examinations (Mindray M7 with P12-4s, 4.2–11 MHz transducer, Mindray Bio-Medical Electronics Co., Shenzhen China), as described previously (Supplementary Fig. 1)^7^. The TTE was performed on a conscious animal (without any drug administration) immobilized manually in a supine position on the dorsum on day 0 (prior to intraperitoneal injection) and on days + 5, + 10, + 15, + 20, + 24 and then every three days and on the day of animal euthanasia. The first endpoint of this study was (1) early-stage PAH characterized by the first morphological lesions of the right ventricle visible on the TTE of rats (end-diastolic right ventricle free wall thickness > 0.7 mm)^7,21^. A total of 12 animals from the study group that met this criterion and eight time-paired rats from the control group were sacrificed. The second endpoint was (2) end-stage PAH (heart failure secondary to PAH) characterized by clinical signs of right ventricle insufficiency up to end-stage circulatory and respiratory failure, including: (1) dyspnea, defined as increased respiratory effort and alternate respiratory motions of the rat’s thorax and the abdomen, (2) decreased temperature of the lower half of the body, the extremities and the tail, assessed subjectively during physical examination (3), cyanotic eyes (4), significantly decreased physical activity, lethargy. A total of 18 animals with end-stage heart failure and eight time-paired rats from the control group were sacrificed. The remaining rats (n = 18) from the monocrotaline-injected group (n = 48) that did not meet the second endpoint during the experiment (n = 12), did not develop any signs of PAH (n = 4), or died under uncontrolled conditions (n = 2) were excluded from the study.

### Hemodynamic examination

On the day of sacrifice, rats were subjected to invasive hemodynamic evaluation. Animals were anesthetized with pentobarbital sodium (30 mg/kg body weight, Biowet, Poland), which was administered intraperitoneally and mechanically ventilated during the whole procedure using a pressure-controlled respirator with a mixture of air and oxygen (80–90%) with isoflurane (2–2.5%, Baxter, Poland) to maintain the anesthesia. If necessary, additional bolus doses of pentobarbital sodium were administered. After infiltration of the surgical site with lidocaine (20 mg/mL, B. Braun Melsungen AG, Germany), the chest cavity was opened via left and right mini thoracotomy at the sixth intercostal space. Next, two heparinized 21G venous cannulas connected to a pressure-recording system were inserted in the right and left ventricles via their apexes to measure systolic and diastolic blood pressure (Siemens SC 7000, Erlangen, Germany)^22^.

### Animal euthanasia and dissection

Rats were sacrificed after hemodynamic examination through an overdose of sodium pentobarbital via intraperitoneal administration. Directly after confirming the termination of vital functions, the chest cavity was fully opened. Blood samples were taken by aortic puncture and collected in tubes with K2-EDTA anticoagulant (Sarsted, Germany). Then, the heart was infused using large volumes of Ringer’s solution (Fresenius Kabi, Germany) to clean the myocardium of blood. Next, using a stereoscopic microscope, the heart was dissected and blot dried.

The tissues of the left and right ventricles’ free walls and interventricular septa were completely separated from each other and the remaining heart structures. The free walls were then weighed and measured. Finally, samples were divided into adequately large sections and frozen in isopentane that was cooled using liquid nitrogen. They were then stored at − 80 °C or fixed in 10% buffered paraformaldehyde solution until subsequent analyses. Blood samples were centrifuged, and plasma was aliquoted and frozen at − 80 °C.

### Selection of study material

Apart from its pneumotoxic action, monocrotaline has also been shown to have direct cardiotoxic effects expressed as myocarditis. Direct monocrotaline myocardial toxicity may lead to heart failure, which may distort the image of characteristic lesions of pulmonary hypertension only^23^. To evaluate signs of local tissue inflammation, histological processing was performed. Paraformaldehyde-fixed samples were dehydrated in a series of alcohols, cleared in xylene, and embedded in paraffin blocks. Samples were cut into 6-µm sections (Leica RM2146 microtome, Germany) and stained with hematoxylin and eosin (Sigma-Aldrich, Germany). In samples with myocarditis caused by monocrotaline, the inflammatory cell infiltration was assessed semi-quantitatively (0 = lack, 1 = low, 2 = moderate, 3 = high, 4 = severe) with a light microscope (Nikon E600, Japan) to eliminate some of them from further analysis. Only samples with less than moderate signs of myocarditis were processed and subjected to further analyses.

Among the rats that developed early PAH (n = 12 animals), none showed signs of local tissue inflammation. A total of eight samples were randomly selected from this cohort for further analyses and matched with eight time-paired rats from the control group. Among the 18 animals with end-stage heart failure, five showed significant signs of myocarditis. Eight samples were randomly selected from the end-stage cohort for further analyses and matched with eight time-paired rats from the control group. As a result, we have distinguished the following four groups (n = 8 each): early PAH rats (group I), control rats time-paired with early PAH animals (group II), end-stage PAH rats (group III), and control rats time-paired with end-stage PAH animals (group IV).

### Determination of plasma markers

The concentration of selected substances in the plasma of sacrificed rats was determined using an enzyme-linked immunosorbent assay (ELISA) according to the manufacturers’ instructions. The following substances were investigated:

- N-terminal pro-B-type natriuretic peptide (NT-proBNP) as a surrogate marker for heart failure severity (Rat NT-proBNP ELISA Kit, LifeSpan BioSciences, US)
- Serpin peptidase inhibitor, a clade A member 3 (SERPINA3) that acts as an inhibitor of various serine proteases (mainly cathepsin G) released during the inflammation and involved in tissue degradation. High levels are associated with poor clinical outcome in heart failure (Rat Serpina3n ELISA Kit, LifeSpan BioSciences, US).
- Endothelin-1, a key mediator in the pathogenesis of PAH (Endothelin-1 Quantikine ELISA Kit, Bio-Techne, US)
- Interleukin-6, an inflammatory cytokine that promotes the development and progression of pulmonary vascular remodeling and heart failure (Rat Interleukin-6 ELISA Kit, Sigma, Germany)

### Autophagy assessment

Marker proteins of the autophagy process were analyzed using a western blot technique. Protein was isolated from 32 left ventricle samples using a Signal Seeker Ubiquitin Enrichment Kit (Cytoskeleton, CO, USA). The obtained protein lysates (40 µg) were then denatured with 2× Laemmli loading buffer (Biorad, CA, USA) at 95 °C for 5 min. SDS-PAGE electrophoresis was performed on 4–20% gradient gel, followed by transfer of proteins from the gel to a polyvinylidene difluoride (PVDF) membrane (Biorad, CA, USA). Membranes were blocked with 5% skim milk dissolved in TBST buffer overnight at 4 °C.

Incubation with primary antibodies against LC3B proteins (dilution 1:1000, Cell Signaling, MA, USA) was carried out overnight at 4 °C. After washing, membranes were incubated with appropriate secondary antibodies (dilution 1:5000, Abcam, United Kingdom) conjugated with horseradish peroxidase for 2 h in room temperature. After washing, ECL substrate (Biorad, CA, USA) was added, and the chemiluminescence signal was detected with an EC3 Imaging System (UVP, CA, USA). Beta-actin was used as an endogenous reference protein. Hela lysate chloroquine treated and HELA lysate untreated (NOVUS Biologicals, CO, USA) were used as positive and negative controls for the autophagy process, respectively. The dedicated software of the EC3 Imaging System (UVP, CA, USA) was used for densitometric analysis.

Immunofluorescent staining was performed after mounting frozen samples on a cryostat chuck with a few drops of tissue-freezing medium (Tissue Tek; Sakura Finetek Europe, Zoeterwoude, The Netherlands). Serial sections (10 μm thick) were cut at − 20 °C in a cryostat (Slee MEV, Mainz, Germany), and frozen sections were fixed in methanol and incubated for 30 min in 5% normal goat serum. They were then incubated overnight at 4 °C with the primary antibody, anti-LC3B polyclonal antibody (dilution 1:200, Cell Signaling, 2775).

After several washes in 0.01 M sodium phosphate buffer containing 0.05% Triton-X, sections were incubated overnight at 4 °C with goat anti-mouse secondary antibodies conjugated with Alexa Fluor 488 (dilution 1:1000, Thermo Fisher Scientific A11001) and goat ant-rabbit secondary antibodies conjugated with Alexa Fluor 555 (dilution 4:1000, Thermo Fisher Scientific A21428). After final washing, preparations were mounted in Vectashield with DAPI medium (Vector Labs, Burlingame, CA, USA) and examined with a laser confocal scanning microscope (FluoView1200, Olympus, Japan) to detect the increase in point collection of tagged LC3B protein. Samples were blindly scored as having low, moderate, or high LC3 puncta abundance by assessing 5 different fields for each left ventricular sample.

### Ubiquitin-dependent proteolysis process assessment

To assess the intensity of ubiquitin-dependent proteolysis process in the left ventricle, ubiquitinated protein content in 32 left ventricle samples was quantitatively analyzed. First, ubiquitinated proteins were immunoprecipitated performed using a Signal Seeker Ubiquitin Enrichment Kit (Cytoskeleton, CO, USA). The obtained protein fractions were subjected to the western blot procedure as described above. All ubiquitinated proteins on PVDF membranes were detected using horseradish peroxidase conjugated anti-ubiquitin antibodies (dilution 1:2000, Precision Red Advanced Protein Assay, Signal Seeker Ubiquitin Enrichment Kit, Cytoskeleton, CO, USA). After the addition of the ECL substrate (Biorad, CA, USA), the chemiluminescent signal was read with an EC3 Imaging System (UVP, CA, USA), and densitometric analysis was performed.

### Statistical analyses

Data were analyzed using StatSoft STATISTICA 13.5 software for Windows (StatSoft Inc, Tulsa, OK). The data are presented as mean values with the corresponding standard deviations (SDs). The Shapiro–Wilk test was used to determine whether quantitative data were normally distributed. Comparisons were performed using a t-test or Mann–Whitney test depending on the normality of the distributions. One-way ANOVA with Tukey’s post hoc tests were performed on log-transformed data. A *p* value lower than 0.05 was considered statistically significant.

### Ethical approval

This study was approved by the 2nd Local Ethical Committee in Cracow, Poland (No. 60/2016) and was performed in accordance to the guidelines from Directive 2010/63/EU of the European Parliament on the protection of animals used for scientific purposes.

## Results

### Echocardiographic, hemodynamic, and morphometric parameters

The mean experiment time (from day 0 to euthanasia) in the early PAH group was 22.0 ± 1.5 days, while it was 37.0 ± 7.1 days in the end stage-PAH group. Echocardiographic, hemodynamic, and morphometric parameters obtained on the days of sacrifice are presented in Table 1. The in vivo echocardiographic examination showed significant thickening of the right ventricle free wall starting from the early PAH stage (early PAH: 0.76 ± 0.07 vs. 0.59 ± 0.09 mm, *p* < 0.001; end-stage PAH: 1.09 ± 0.09 vs. 0.64 ± 0.05 mm, *p* < 0.001). The echocardiographic image of the left ventricle showed no difference between early PAH and matched control rats (end-diastolic left ventricular free wall thickness: 2.13 ± 0.61 vs. 2.02 ± 0.82 mm, *p* = 0.781).

**Table 1:** Echocardiographic, hemodynamic and morphometric parameters measured at euthanasia day (mean ± SD).

Parameter	early PAH rats (n = 8)	non-PAH matched control rats (n = 8)	*p* value	end-stage PAH rats (n = 8)	non-PAH matched control rats (n = 8)	*p* value
Echocardiographic measurements						
RVFWTd (mm)	0.76 ± 0.07	0.59 ± 0.09	< **0**.**001**	1.09 ± 0.09	0.64 ± 0.05	< **0**.**001**
LVFWTd (mm)	2.13 ± 0.61	2.02 ± 0.82	0.781	1.84 ± 1.10	2.16 ± 0.65	0.509
TAPSE (mm)	1.10 ± 0.17	1.40 ± 0.45	0.099	0.72 ± 0.19	1.31 ± 0.48	**0**.**006**
PAAT/CL	0.21 ± 0.05	0.23 ± 0.08	0.558	0.16 ± 0.04	0.23 ± 0.07	**0**.**027**
Hemodynamic measurements						
RV systolic pressure (mmHg)	27.80 ± 4.53	19.20 ± 6.02	**0**.**005**	53.10 ± 13.30	23.80 ± 3.61	< **0**.**001**
RV diastolic pressure (mmHg)	9.13 ± 1.82	6.21 ± 2.91	**0**.**03**	4.82 ± 2.75	6.16 ± 2.51	0.326
LV systolic pressure (mmHg)	88.22 ± 9.98	90.50 ± 14.21	0.715	44.28 ± 11.93	91.71 ± 14.84	< **0**.**001**
LV diastolic pressure (mmHg)	8.90 ± 2.45	11.21 ± 5.03	0.262	5.74 ± 3.86	10.31 ± 5.83	0.086
Morphometric measurements						
Body weight at euthanasia day (g)	366.5 ± 21.9	375.3 ± 63.3	0.715	352.8 ± 32.3	389.9 ± 72.3	0.206
Body weight change (0 day—euthanasia day) (g)	44.6 ± 8.7	53.3 ± 27.9	0.589	37.4 ± 26.4	68.6 ± 25.3	**0**.**038**
RV free wall weight (g)	0.22 ± 0.04	0.18 ± 0.02	**0**.**024**	0.36 ± 0.07	0.17 ± 0.05	< **0**.**001**
LV free wall weight (g)	0.35 ± 0.04	0.38 ± 0.08	0.358	0.24 ± 0.04	0.38 ± 0.09	**0**.**001**
RV free wall thickness (mm)	0.87 ± 0.19	0.57 ± 0.21	**0**.**001**	1.13 ± 0.20	0.60 ± 0.14	< **0**.**001**
LV free wall thickness (mm)	2.41 ± 0.48	2.30 ± 0.45	0.643	1.89 ± 0.53	2.39 ± 0.54	0.082

LV, left ventricle; LVFWTd, end-diastolic left ventricular free wall thickness; PAAT/CL, pulmonary artery acceleration time normalized to cycle length; PAH, pulmonary arterial hypertension; RV, right ventricle; RVFWTd, end-diastolic right ventricular free wall thickness; TAPSE, tricuspid annular plane systolic excursion.

Statistically significant *p* value s are given in bold.

Furthermore, the end-stage PAH group showed a noticeable but statistically insignificant reduction of the echocardiographically assessed end-diastolic wall thickness of the left ventricle in the end-stage PAH group (1.84 ± 1.10 vs. 2.16 ± 0.65 mm, *p* = 0.509). The measured TAPSE and PAAT/CL parameters indicated development of significant pulmonary hypertension in the end-stage PAH group (Table 1). The measured hemodynamic parameters showed a gradually increasing right ventricular pressure load with PAH progression (early PAH group right ventricular systolic pressure: 27.80 ± 4.53 vs. 19.20 ± 6.02 mmHg, *p* = 0.005; end-stage PAH group: 53.10 ± 13.30 vs. 23.80 ± 3.61 mmHg, *p* < 0.001). Moreover, significantly impaired left ventricular systolic function was noticed in the end-stage PAH group (44.28 ± 11.93 vs. 91.71 ± 14.84 mmHg, *p* < 0.001). No such deviation was noted in the early stage of PAH development (88.22 ± 9.98 vs. 90.50 ± 14.21 mmHg, *p* = 0.715) (Table 1).

A significant increase in right ventricle myocardium mass was detected during autopsy in both early and end-stage PAH rats (0.22 ± 0.04 vs. 0.18 ± 0.02 g, *p* = 0.024 and 0.36 ± 0.07 vs. 0.17 ± 0.05 g, *p* < 0.001, respectively). This was accompanied by visible thickening of the right ventricular myocardium (early PAH: 0.87 ± 0.19 vs. 0.57 ± 0.21 mm, *p* = 0.001, end-stage PAH: 1.13 ± 0.20 vs. 0.60 ± 0.14 mm, *p* < 0.001). On the other hand, a significant decrease in left ventricle myocardium mass in the end-stage PAH group was detected when compared to the controls (0.24 ± 0.04 vs. 0.38 ± 0.09, *p* = 0.001) (Table 1).

### PAH and heart failure plasma markers

**Figure 1:**
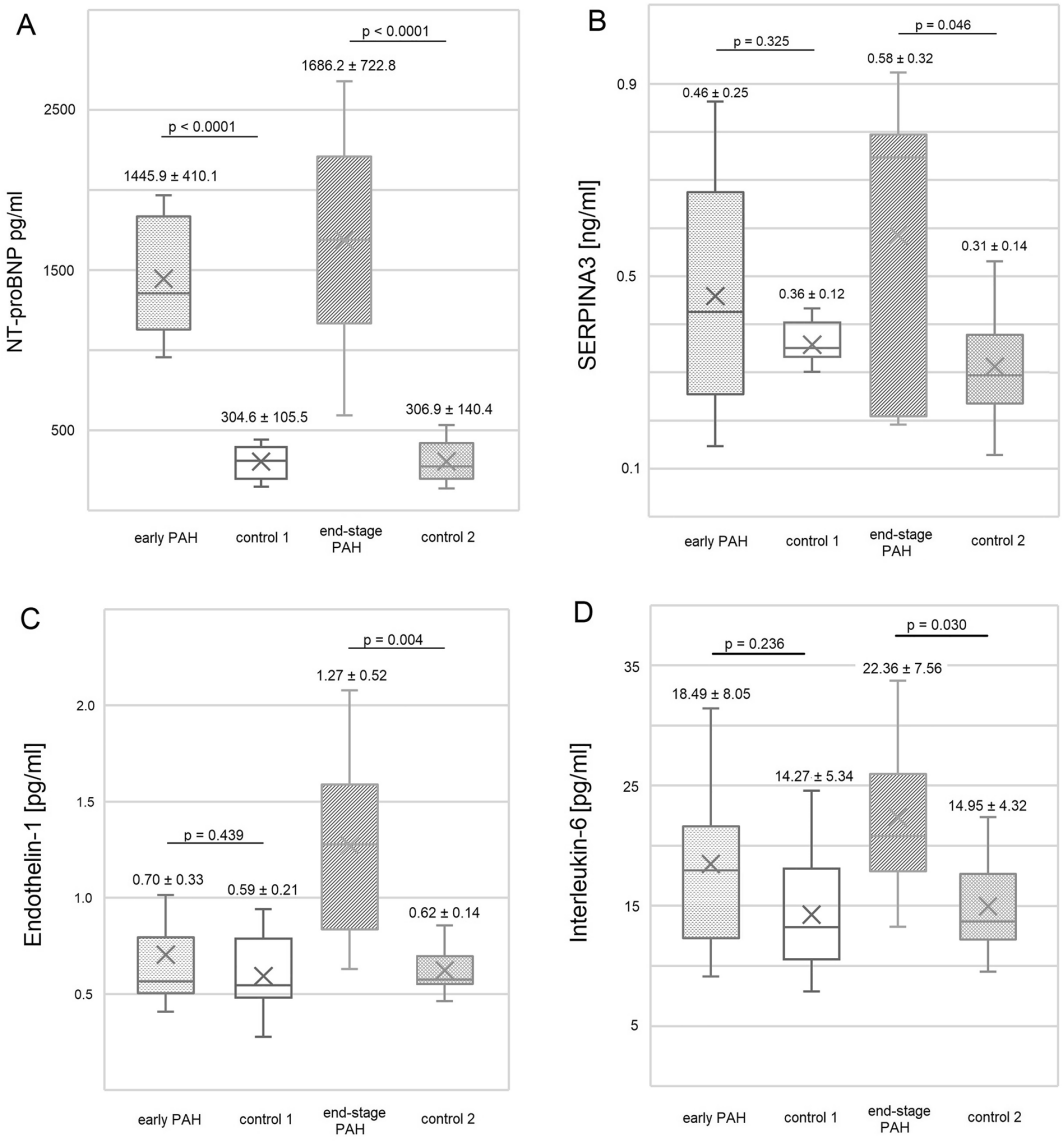
Box and whisker plots comparing plasma levels of (**A**) NT-proBNP, (**B**) SERPINA3, (**C**) Endothelin-1 and (**D**) Interleukin-6 in early PAH, end-stage PAH and matched controls. Data are presented as mean ± SD, n = 8 per group. Statistical analyses were performed using a t-test or Mann–Whitney test depending on the normality of the distributions. Control 1—control rats time-paired with early PAH animals, control 2—control rats rats time-paired with end-stage PAH animals.

A significant increase in the plasma concentration of NT-proBNP was discovered during the course of PAH, confirming the development of severe heart failure (*p* < 0.0001, Fig. 1A). Concentrations of the other three analyzed plasma factors, SERPINA3, endothelin-1, and interleukin-6, showed no statistically significant change at an early PAH stage compared to the controls (all *p* > 0.05, Figs. 1 B-D). Nevertheless, samples collected from end-stage PAH rats showed substantially higher concentrations of SERPINA3, endothelin-1, and interleukin-6 compared to healthy animals (all *p* < 0.05) (Fig. 1B–D).

### Autophagy process assessment by western blotting

**Figure 2:**
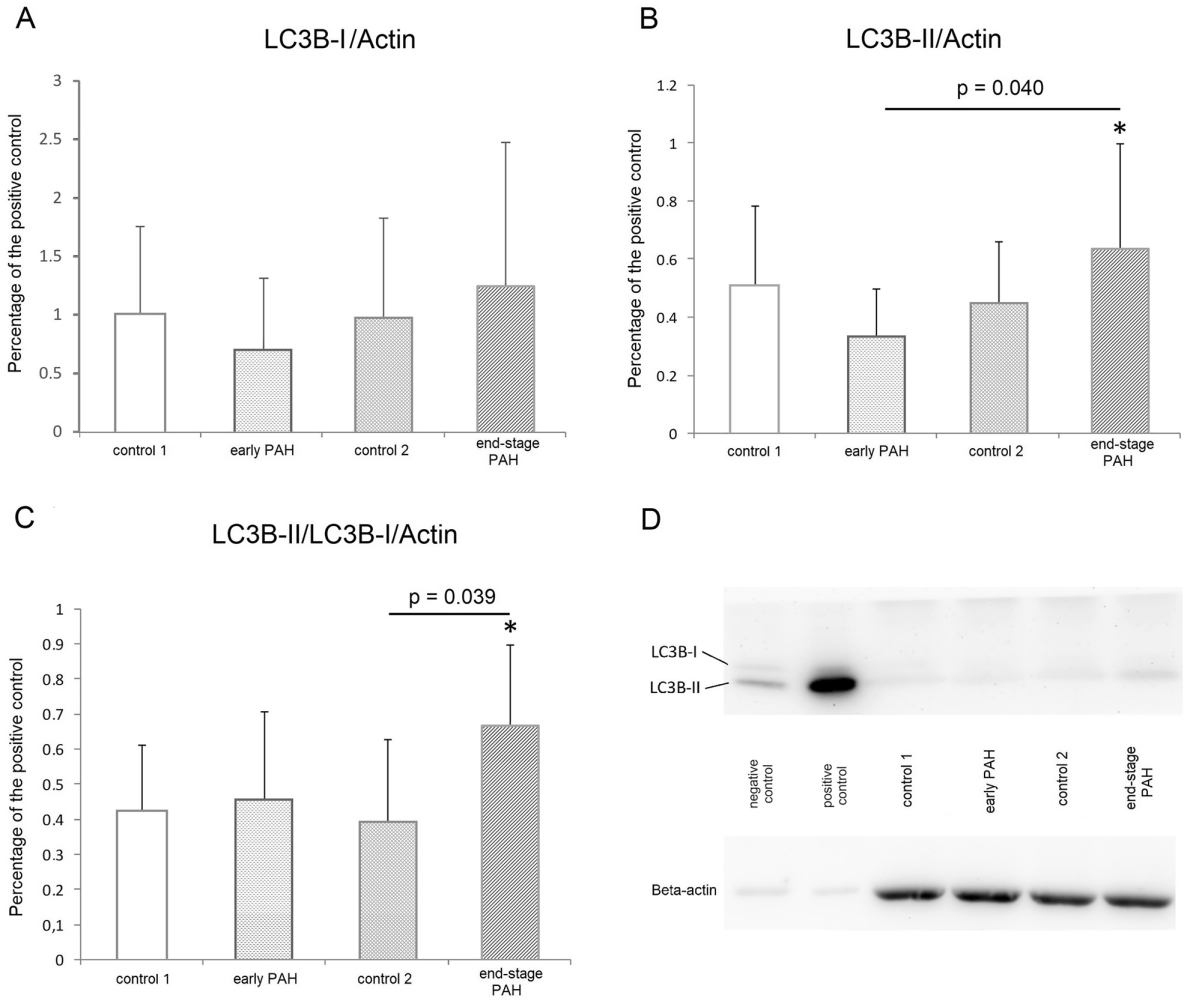
Left ventricle myocardium autophagy process assessment by Western blotting. (**A**) Cardiac LC3B-I expression evaluated by Western blotting and expressed relative to beta-actin content in early PAH, end-stage PAH and matched controls. (**B**) Cardiac LC3B-I expression evaluated by Western blotting and expressed relative to beta-actin content in early PAH, end-stage PAH and matched controls. (**C**) LC3B-II/LC3B-I/beta-actin ratio evaluated by Western blotting in all analyzed groups. (**D**) Representative Western blot showing the expression of LC3BI/II in all analyzed groups (cropped blots); original blots are presented in Supplementary Figs.  2–5. N = 8 per group. The results were calculated relative to the positive control, assuming that the expression of positive control was 100%. Statistical analyses were performed with one-way ANOVA and Tukey post hoc test; significance: *p < 0.05 end stage PAH versus early PAH in LC3B-II protein expression, *p < 0.05 end stage PAH versus control 2 in LC3B-II/LC3B-I ratio. Control 1—control rats time-paired with early PAH animals, control 2—control rats rats time-paired with end-stage PAH animals.

We determined LC3B-I and LC3B-II levels by performing western blot analysis of the left ventricle myocardium from PAH rats and matched controls (Fig. 2). The results for LC3 were quantitatively analyzed using beta-actin as the normalizing protein (Fig. 2D). LC3 was detected as two bands: cytosolic LC3B-I and membrane LC3B-II. Autophagy can be assessed by tracking the level of LC3B-I to LC3B-II conversion.

No statistically significant difference in LC3B-I expression was noted in early the PAH group versus healthy controls, the end-stage PAH group versus healthy controls, and the early PAH group versus end-stage PAH (Fig. 2A). On the other hand, the LC3B-II expression in the left ventricle myocardium samples was significantly higher in end-stage PAH rats compared to the early PAH group (Fig. 2B) (*p* = 0.040). Moreover, LC3B-II/LC3B-I ratios in end-stage PAH samples were significantly elevated compared to those in healthy controls (Fig. 2C) (*p* = 0.039). In the context of autophagy, an increase in LC3B-II levels or a higher LC3B-II/LC3B-I ratio indicates an upregulation of autophagic activity.

### Histological analysis

**Figure 3:**
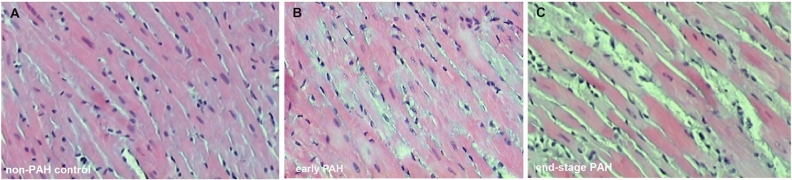
Representative histological images of the left ventricle myocardium. Histological cross-sections of left ventricle samples stained with hematoxylin and eosin (H&E) from (**A**) non-PAH control group (**B**) early PAH and (**C**) end-stage PAH group. Left ventricle paraformaldehyde-fixed samples were dehydrated in a series of alcohols, cleared in xylene, and embedded in paraffin blocks. Samples were cut into 6-µm sections (Leica RM2146 microtome, Germany) and stained with hematoxylin and eosin (Sigma-Aldrich, Germany).

**Figure 4:**
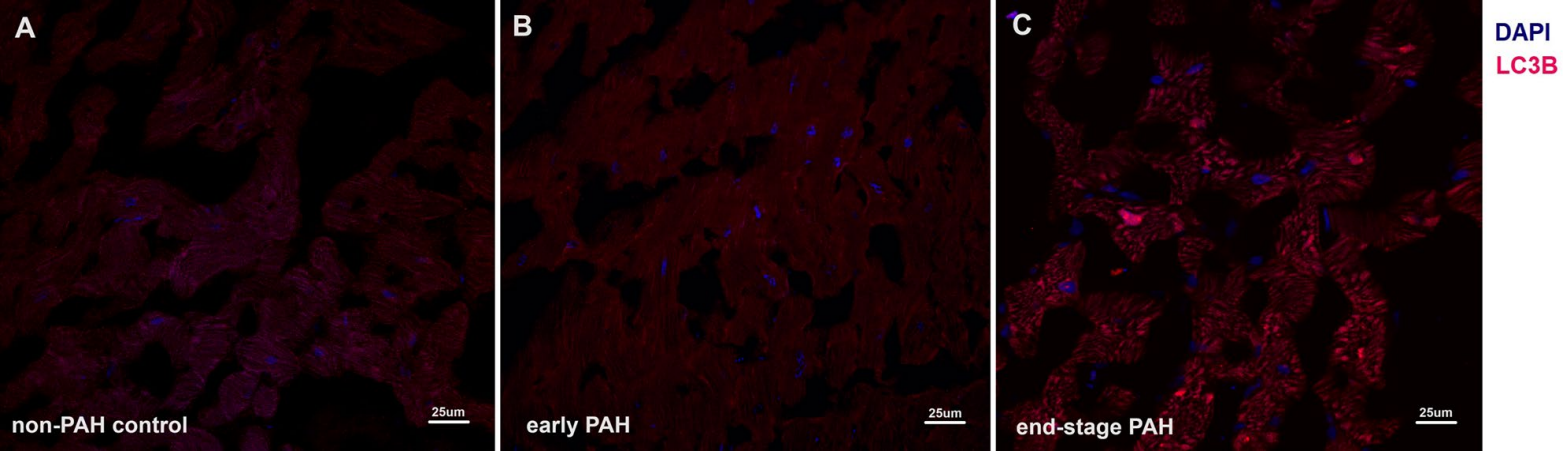
Representative immunofluorescence images showing abundance of LC3 in left ventricle from (**A**) non-PAH control group (low LC3 puncta abundance) and (**B**) early PAH and (**C**) end-stage PAH group (high LC3 puncta abundance). Immunofluorescent staining was performed on frozen left ventricle samples; anti-LC3B polyclonal antibody (dilution 1:200, Cell Signaling, 2775), goat anti-mouse secondary antibodies conjugated with Alexa Fluor 488 (dilution 1:1000, Thermo Fisher Scientific A11001) and goat ant-rabbit secondary antibodies conjugated with Alexa Fluor 555 (dilution 4:1000, Thermo Fisher Scientific A21428), DAPI medium (Vector Labs, Burlingame, CA, USA).

Hematoxylin and eosin staining of samples showed considerable structural changes of the left ventricular myocardium in end-stage PAH subjects (Fig. 3). Reduction in the size of cardiomyocytes and increased connective tissue volume were observed in end-stage PAH animals compared to both non-PAH controls and the early PAH group (Fig. 3). Anti-LC3B immunofluorescence staining was performed to assess the autophagic response in samples (Fig. 4). A significant difference in the abundance of LC3 puncta was detected when comparing the left ventricle in the end-stage PAH group (all samples had high LC3 puncta abundance, Fig. 4C) to both matched controls (all samples had low abundance or none, Fig. 4A) and early-PAH subjects (75% of samples had low abundance, and 25% had moderate abundance, Fig. 4B).

### Ubiquitin-dependent proteolysis process in left ventricle

**Figure 5:**
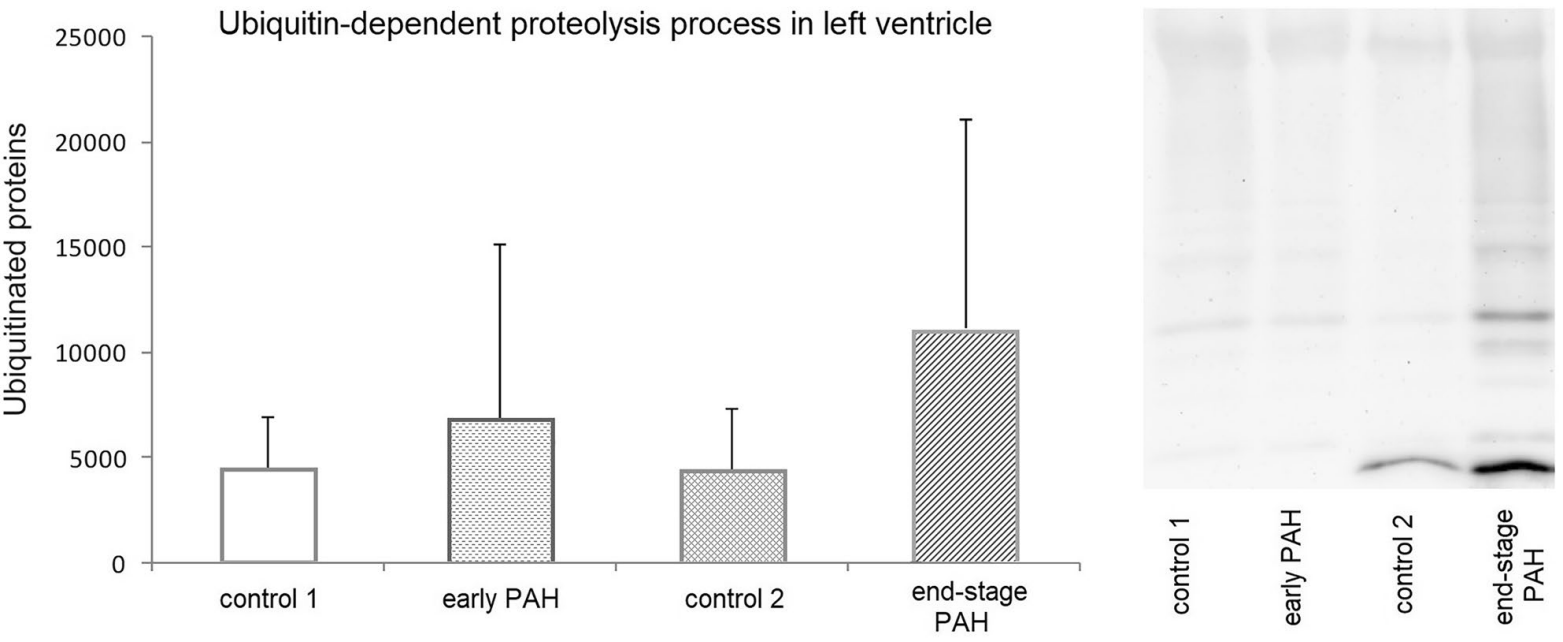
Ubiquitin-dependent proteolysis process in left ventricle assessed by immunoprecipitation of ubiquitinated proteins. Levels of expression of ubiquitinated proteins normalized to 1 mg of protein used for immunoprecipitation in early PAH, end-stage PAH and matched controls. N = 8 per group. Statistical analyses were performed with one-way ANOVA and Tukey post hoc test. Representative Western blot showing the expression of ubiquitinated proteins in all analyzed groups (cropped blot); original blots are presented in Supplementary Figs. 2–5. Control 1—control rats time-paired with early PAH animals, control 2—control rats rats time-paired with end-stage PAH animals.

There was no statistically significant difference in the levels of expression of all ubiquitinated proteins normalized to 1 mg of protein used for immunoprecipitation comparing both PAH groups and matched controls (ANOVA, *p* = 0.332) (Fig. 5). The mean density area of all ubiquitinated proteins for the end-stage PAH group was 10967.0, which was not statistically different from controls (4424.8; *p* = 0.32) and the early-stage PAH group (6829.1; *p* = 0.631).

## Discussion

Molecular processes underlying left ventricle remodeling over the course of PAH still remain poorly understood. In particular, there is little knowledge regarding the mechanisms of left ventricle atrophy^17,24^. Therefore, we aimed to find molecular mechanisms that may be responsible for the loss of left ventricular myocardial mass over the course of PAH. Our results suggest that autophagy may be considered as one of these at the end stage of PAH, while ubiquitin-dependent proteolysis seems not to play a significant role in this phenomenon. Our previous study has shown that left ventricle atrophy in PAH is linked to an increase in apoptotic pathway activity and intensified fibrosis^17^, which may indicate that there is crosstalk between the degradation mechanisms of autophagy and apoptosis in the left ventricle during the end stage of PAH.

One of the proposed causes of left ventricle atrophy during PAH includes a decrease in the initial load of the left heart (hemodynamic stress). Another possible mechanism of left ventricle mass loss in PAH is related to general hypoxia and ischemia of the myocardium resulting from right ventricle heart failure (metabolic stress)^25^. It has been shown that autophagy plays a significant role in the degradation of cardiomyocytes' cellular components in a model of a hemodynamically unloaded heart. In contrast, a failing human heart already expresses elevated levels of autophagy markers, but when implanted with a left ventricular assist device, autophagy markers are downregulated^25^. This indicates that autophagy participates in the processes of the myocardium’s response to conditions of hemodynamic load, which may also occur in the case of PAH.

Apoptosis and autophagy are significant processes for the correct development of organisms and decisive effects on cell destinies. Numerous studies show that various stimuli are capable of activating both autophagy and apoptosis, and despite the clear differences between these two processes, their regulation at the molecular level is related^26,27^. In the context of cell death, it is possible to distinguish two major types of interactions between autophagy and apoptosis. In the first one, autophagy and apoptosis are partners operating in a coordinated and cooperative manner. In this case, autophagy and apoptosis act together or as two independent pathways and may lead to cell elimination. In the second mechanism, autophagy enables the process of apoptosis. In such a scenario, autophagy does not lead to cellular death per se, but it mediates inductions of death in the process of apoptosis^28^.

Our study also provides several other important observations regarding the left ventricular remodeling during PAH. First, significantly impaired left ventricular systolic function occurred in the end-stage PAH group, but no such change was visible in the early-stage PAH group (Table 1). Considerable echocardiographic and morphological remodeling of the left ventricle was also observed over the course of monocrotaline-induced PAH (Table 1). Moreover, at the end stage of PAH, the left ventricle myocardium was more fibrotic, and the cardiomyocyte size was significantly reduced compared to both non-PAH subjects and the early PAH stage. Both a reduction in the size of cardiomyocytes, replacement with less dense, more rigid, and less functional fibrotic tissue, and the loss of myocardial structural proteins^17^ may explain the observed decrease in left ventricle mass. This was accompanied by a significant increase of key plasma markers related to PAH and heart failure, but such an increase was observed late in the final PAH phase (Fig. 1). This paints a picture of considerable left ventricle impairment, which appears at the end stages of PAH secondary to the right ventricular failure.

There are several limitations to the current study, and the main one is that the results of animal experiments may not be fully translated into clinical manifestations of diseases in humans. In this study, we adopted a monocrotaline model of PAH. It is well proven that monocrotaline induces endothelial cell damage resulting in pulmonary vascular remodeling, increased vascular resistance, thus development of PAH, as well as cardiotoxic effects expressed as myocarditis. Direct monocrotaline toxicity with regard to the myocardium may lead to heart insufficiency (regardless of hemodynamic changes caused by PAH), which may distort the image of lesions characteristic of pulmonary hypertension only^23^. To minimize this bias, we implemented a pre-selection protocol that rejected samples with signs of myocarditis, which should have addressed most of the negative influence of monocrotaline in this regard.

Furthermore, it should be emphasized that the development of end-stage heart failure in the PAH model occurs much faster than in the natural course of this disease in humans^20^. Nevertheless, the monocrotaline rat model of PAH is a widely accepted and commonly used experimental model, without which it would not be possible to conduct research of a similar nature^29^. Heart tissue samples from living patients with different PAH stages and healthy controls are unobtainable without a significant risk for the study participants.

Despite the limitations, our study has several important strengths. The main one is that it analyzed not only the final stage of PAH-induced heart failure, but also the left ventricle myocardial changes over the early course of PAH progression. Moreover, we have implemented many different techniques to record PAH-related changes, including echocardiography, hemodynamic measurements, morphometrical assessment, histological processing with immunofluorescence staining, assessment of plasma markers of heart failure, autophagy evaluation by western blotting, and immunoprecipitation-based estimation of ubiquitinated proteins. Finally, the design of our study is consistent with other studies using monocrotaline-induced PAH models, so direct study comparisons are feasible.

## Conclusion

Considerable morphological and hemodynamic remodeling of the left ventricle is observed over the course of monocrotaline-induced PAH. End-stage PAH is associated with significantly impaired left-ventricular systolic function and a substantial decrease in left-ventricle myocardium mass. Autophagy may be considered as the mechanism behind the left-ventricular mass loss at the end stage of the PAH, while ubiquitin-dependent proteolysis does not appear to play an important role in this process. Learning the mechanisms of left ventricle mass loss may constitute an anchor point for new therapies for patients with pulmonary hypertension and significantly expand our knowledge regarding cooperation of various metabolic pathways in myocardium plasticity.

### Supplementary Information

Supplementary Figures.

## Data Availability

All data generated or analysed during this study are included in this published article.
