# Supplementary material for: Autophagy and ubiquitin-dependent proteolysis processes in left ventricular mass loss in pulmonary arterial hypertension
Source: Sci Rep. 2024 Jul 2;14:15133. doi: 10.1038/s41598-024-64950-4 (PMC11220073; doi:10.1038/s41598-024-64950-4)

**Supplementary Figures**

**Supplementary Figure 1** – Representative transthoracic echocardiogram images: (A) apical 4-chamber end-diastolic projection showing structurally unchanged rat heart from the control group; (B) apical 4-chamber end-diastolic projection presenting failing rat heart (end-stage pulmonary arterial hypertension); (C) long-axis parasternal view in M-mode view showing end-diastolic left ventricular free wall thickness (LVFWTd) evaluation; (D) apical 4-chamber showing measurement of end-diastolic right ventricular free wall thickness (RVFWTd); (E) pulmonary artery acceleration time (PAAT) measurement (F) the tricuspid annulus plane systolic excursion (TAPSE) analysis. LV - left ventricle, RV - right ventricle.


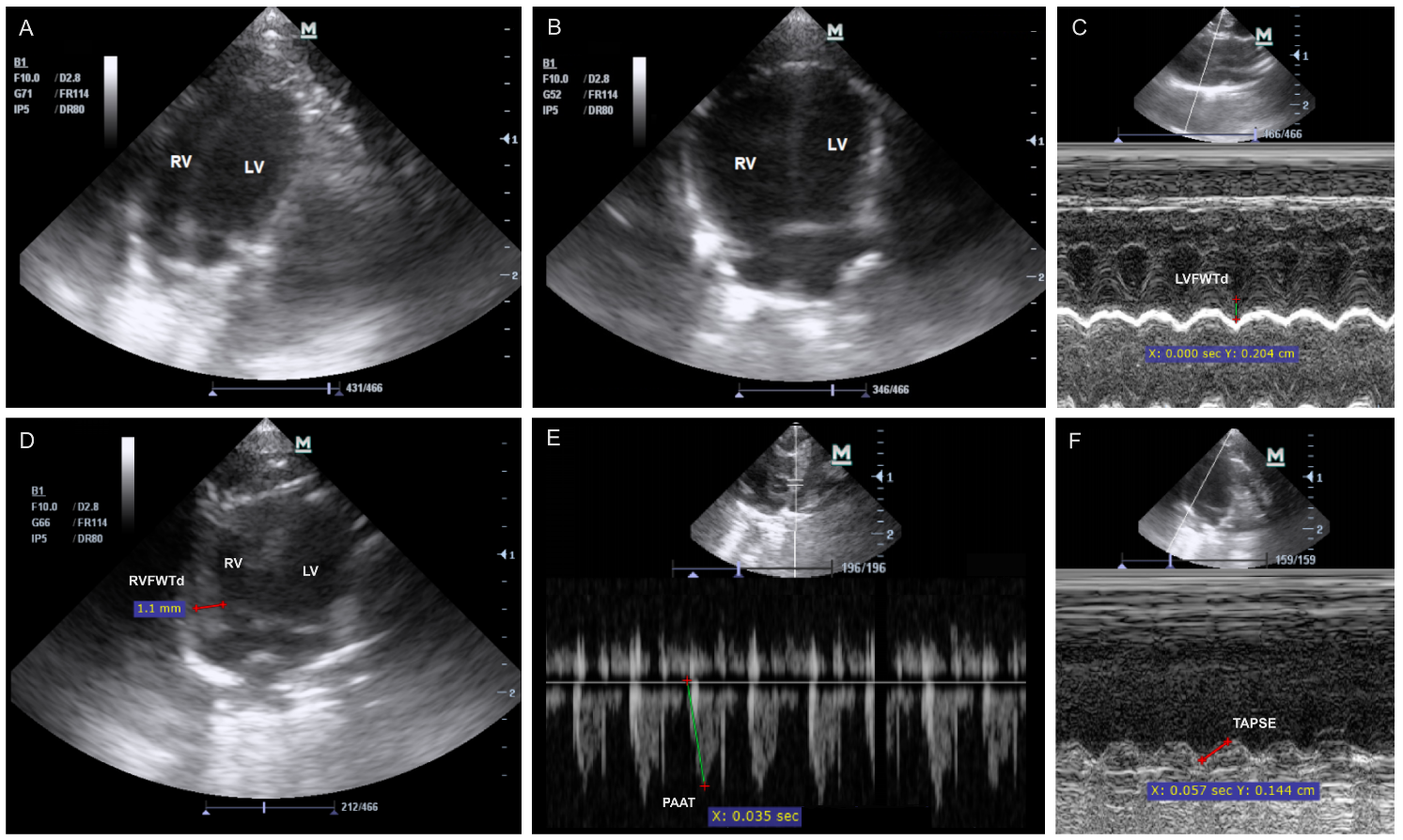


**Supplementary Figure 2 -** Western blot (run 1) showing the expression of A) LC3BI/II, B) beta-actin and C) ubiquitinated proteins in all analyzed groups (original blot).

Regions of the representative blots used in the manuscript were denoted using red boxes.


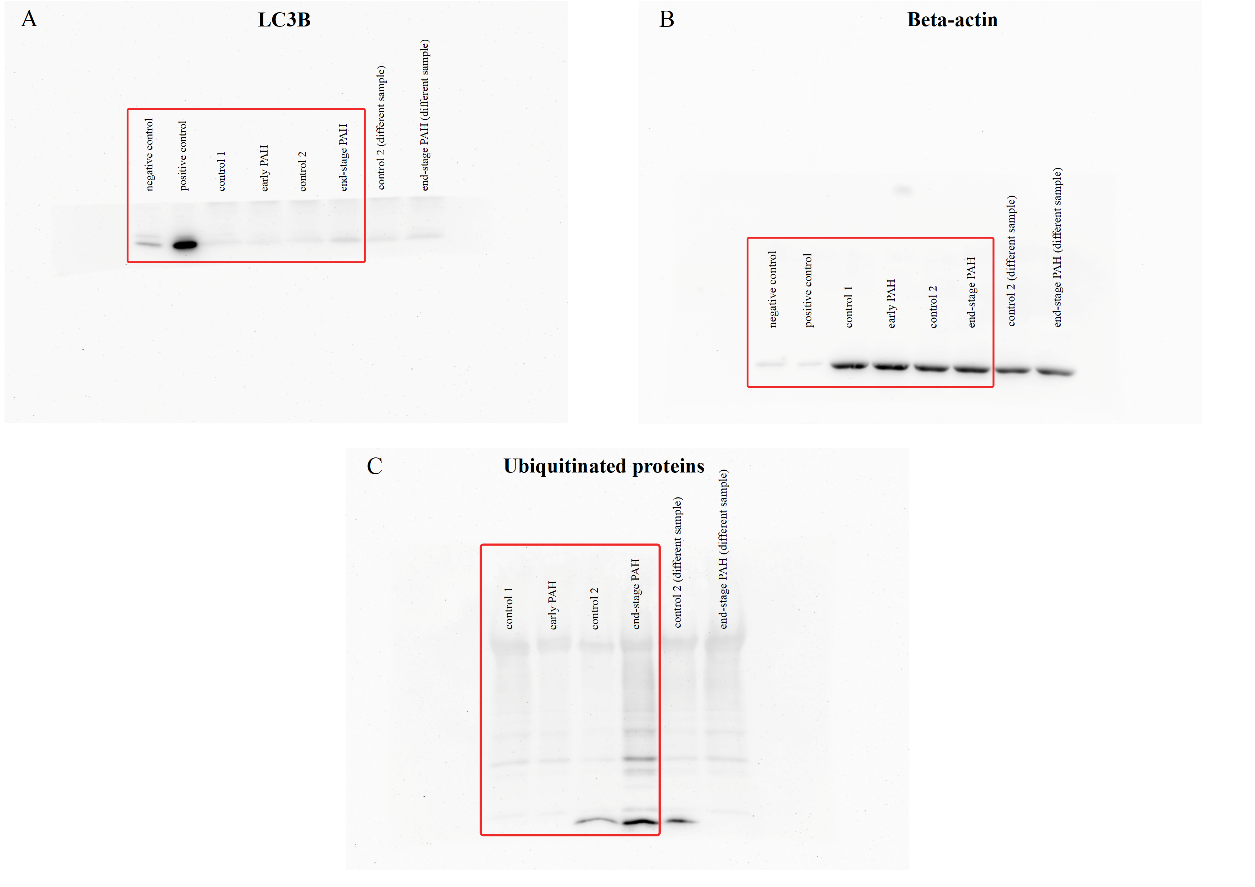


**Supplementary Figure 3 -** Western blot (run 2) showing the expression of A) LC3BI/II, B) beta-actin and C) ubiquitinated proteins in next samples of analyzed groups (original blot).
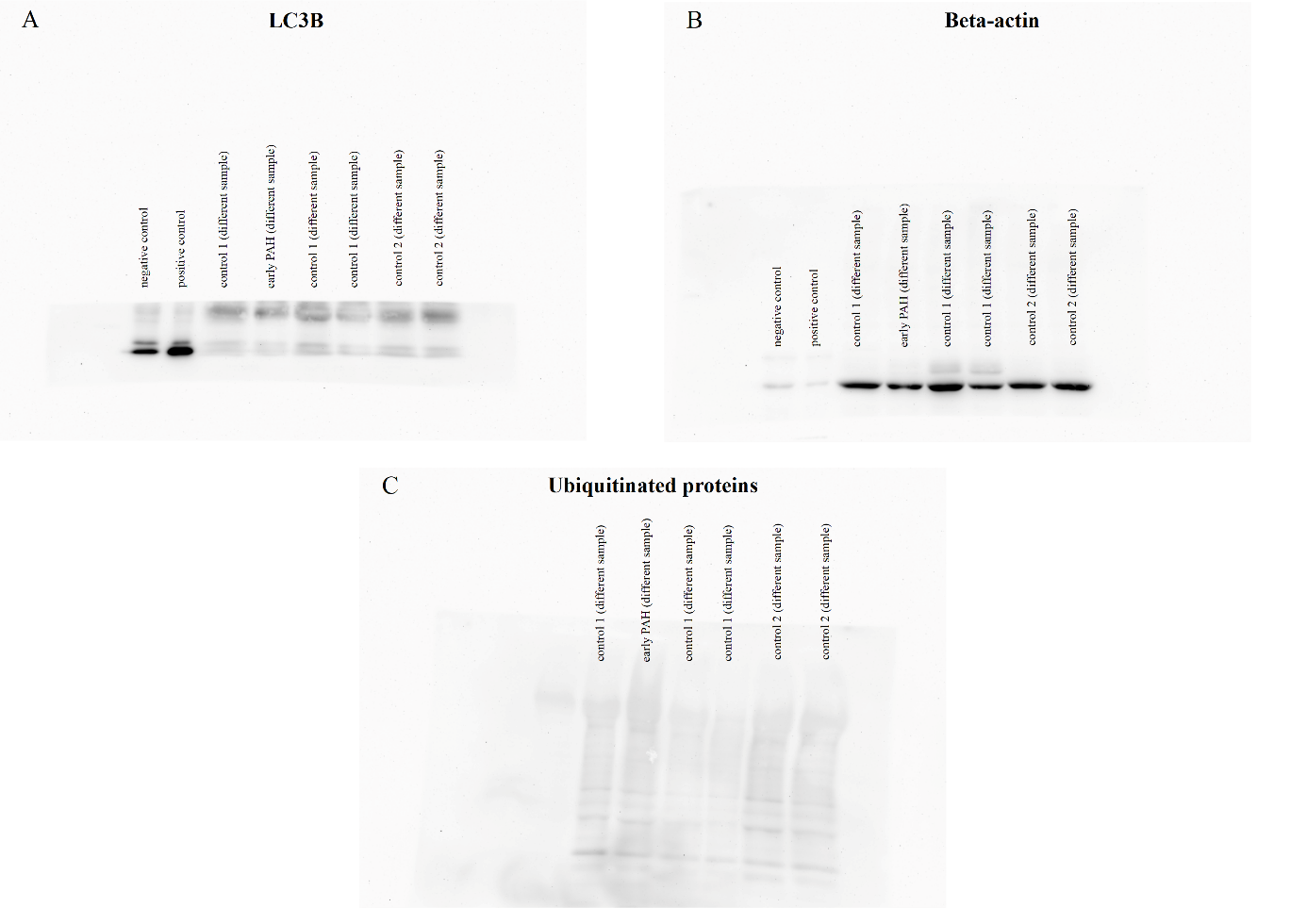


**Supplementary Figure 4 -** Western blot (run 3) showing the expression of A) LC3BI/II, B) beta-actin and C) ubiquitinated proteins in next samples of analyzed groups (original blot).
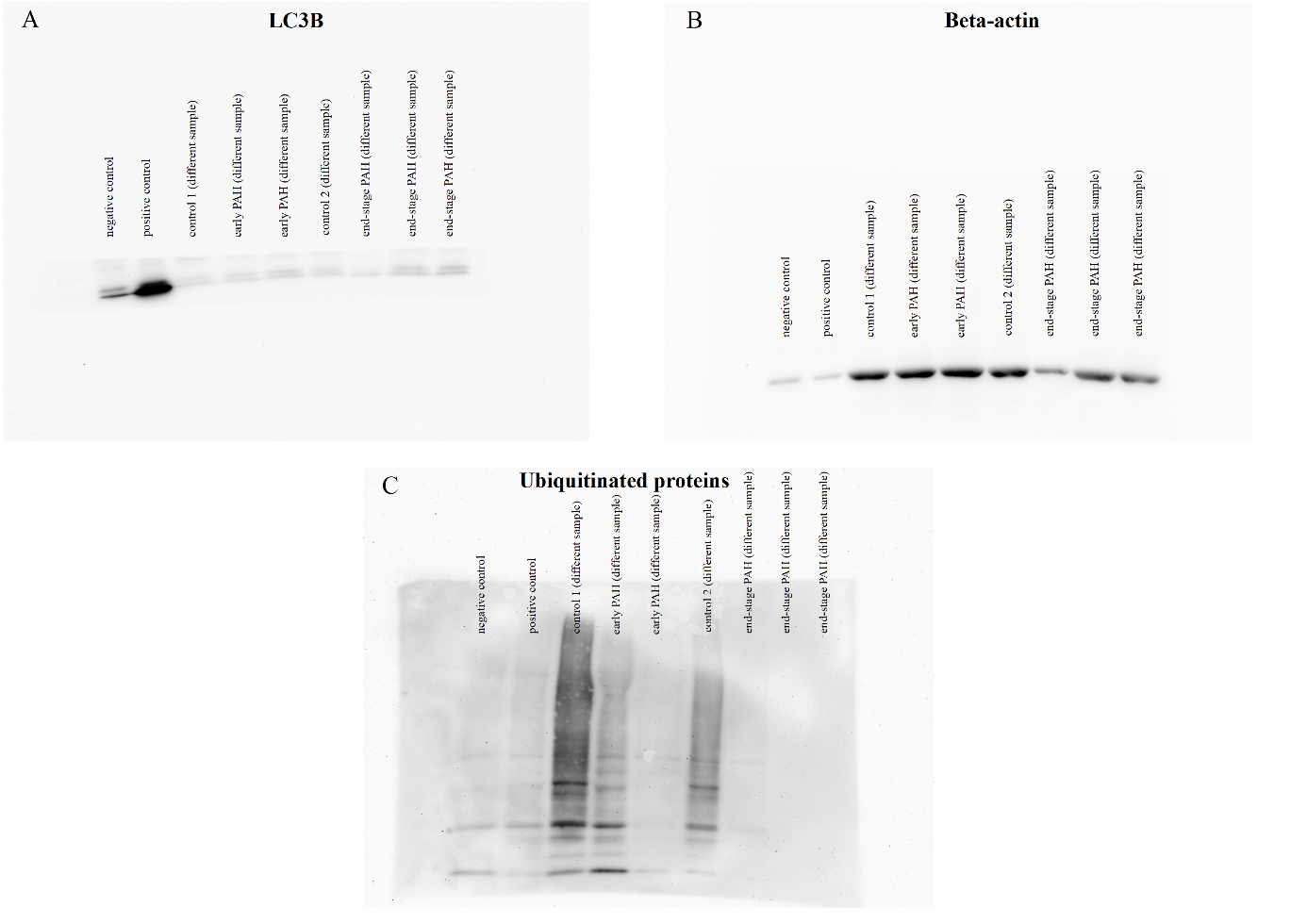


**Supplementary Figure 5 -** Western blot (run 4) showing the expression of A) LC3BI/II, B) beta-actin and C) ubiquitinated proteins in next samples of analyzed groups (original blot).
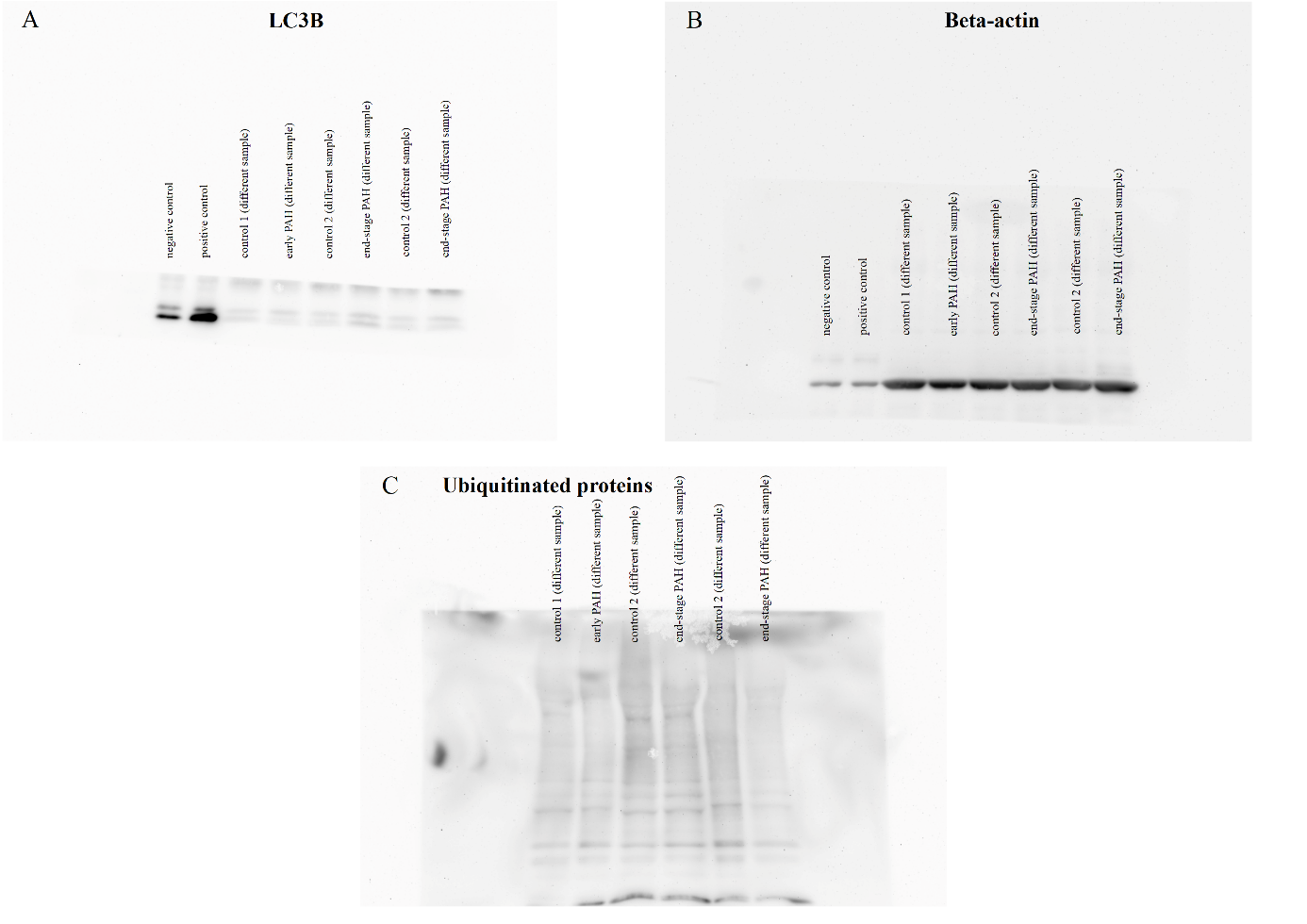


**Supplementary Figure 6 -** Western blot (run 5) showing the expression of A) LC3BI/II, B) beta-actin and C) ubiquitinated proteins in next samples of analyzed groups (original blot).
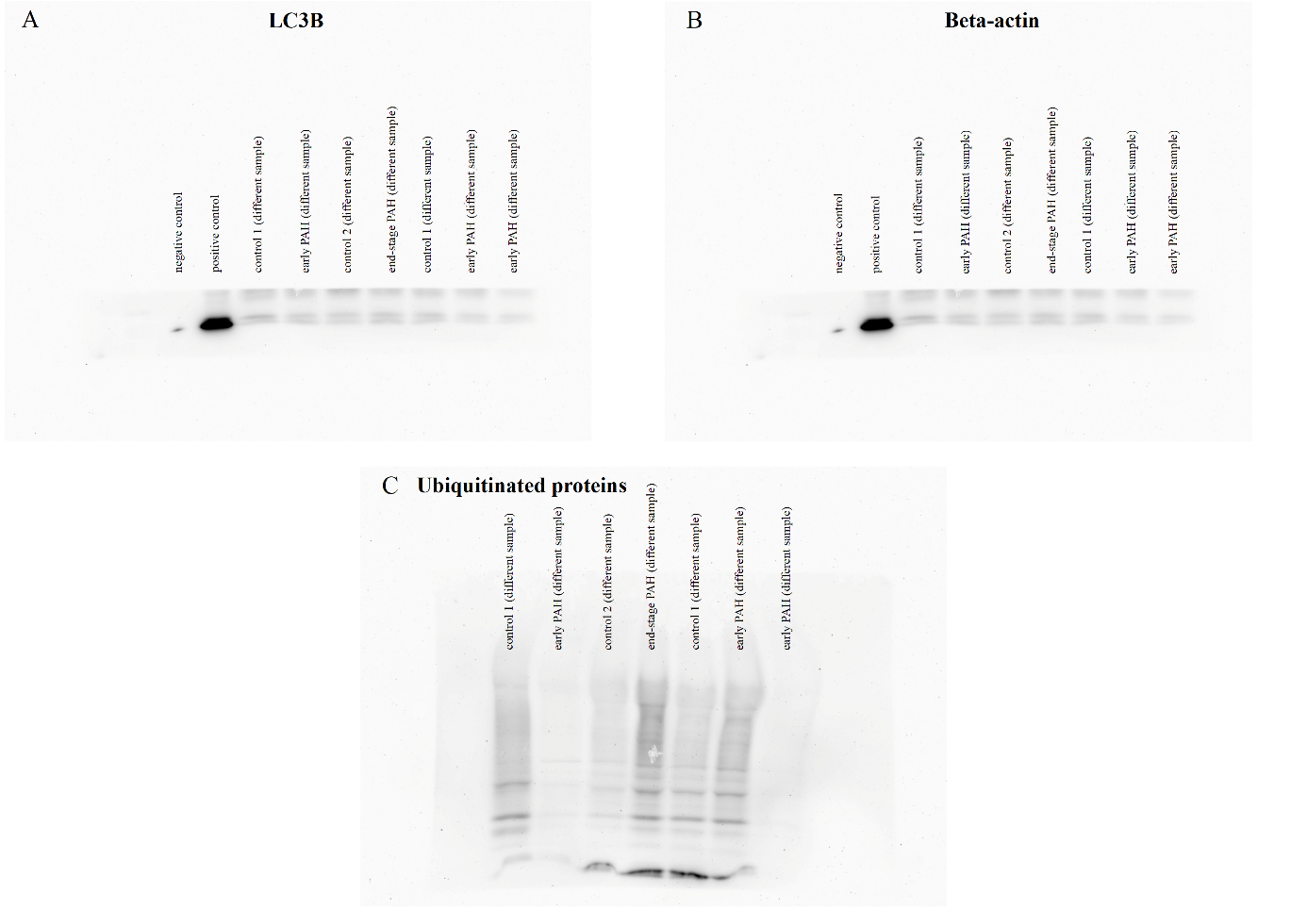

Supplement: Supplementary file 1 — Supplementary Figures. [file 41598_2024_64950_MOESM1_ESM.docx]
